# Investigation of Radiation-Induced Toxicity in Head and Neck Cancer Patients through Radiomics and Machine Learning: A Systematic Review

**DOI:** 10.1155/2021/5566508

**Published:** 2021-06-09

**Authors:** Roberta Carbonara, Pierluigi Bonomo, Alessia Di Rito, Vittorio Didonna, Fabiana Gregucci, Maria Paola Ciliberti, Alessia Surgo, Ilaria Bonaparte, Alba Fiorentino, Angela Sardaro

**Affiliations:** ^1^Radiation Oncology Department, General Regional Hospital “F.Miulli”, Acquaviva Delle Fonti, Bari, Italy; ^2^Radiation Oncology, Azienda Ospedaliero Universitaria Careggi, Florence, Italy; ^3^Radiation Oncology Unit, IRCCS Istituto Tumori “Giovanni Paolo II, Bari, Italy; ^4^Medical Physics Unit, IRCCS Istituto Tumori “GiovanniPaolo II”, Bari, Italy; ^5^Interdisciplinary Department of Medicine, Section of Radiology and Radiation Oncology, University of Bari “Aldo Moro”, Bari, Italy

## Abstract

*Background. *Radiation-induced toxicity represents a crucial concern in oncological treatments of patients affected by head and neck neoplasms, due to its impact on survivors' quality of life. Published reports suggested the potential of radiomics combined with machine learning methods in the prediction and assessment of radiation-induced toxicities, supporting a tailored radiation treatment management. In this paper, we present an update of the current knowledge concerning these modern approaches. Materials and Methods. A systematic review according to PICO-PRISMA methodology was conducted in MEDLINE/PubMed and EMBASE databases until June 2019. Studies assessing the use of radiomics combined with machine learning in predicting radiation-induced toxicity in head and neck cancer patients were specifically included. Four authors (two independently and two in concordance) assessed the methodological quality of the included studies using the Radiomic Quality Score (RQS). The overall score for each analyzed study was obtained by the sum of the single RQS items; the average and standard deviation values of the authors' RQS were calculated and reported. Results. Eight included papers, presenting data on parotid glands, cochlea, masticatory muscles, and white brain matter, were specifically analyzed in this review. Only one study had an average RQS was ≤ 30% (50%), while 3 studies obtained a RQS almost ≤ 25%. Potential variability in the interpretations of specific RQS items could have influenced the inter-rater agreement in specific cases. Conclusions. Published radiomic studies provide encouraging but still limited and preliminary data that require further validation to improve the decision-making processes in preventing and managing radiation-induced toxicities.

## 1. Introduction

Worldwide, head and neck squamous cell carcinoma (HNSCC) represents the sixth most common nonskin cancer, with about 600,000 new cases diagnosed annually [1]. Despite the established role of Human Papilloma Virus (HPV) infection in oropharyngeal cancer [2], a critical lack of prognostic factors limits the possibility to apply personalized medicine in head and neck oncology. Radiotherapy (RT) is an essential component of the aforementioned personalized treatment for HNSCC, however burdened with a high rate of acute and late severe toxicity [3].

The paradigm shift towards quantitative imaging, which has been observed in the last two decades, might represent the turning point to capture individual tumor heterogeneity, along with gene expression profiling [4]. The exponential progress in medical image analysis has allowed an unprecedented, high-throughput extraction of quantitative features. Radiomics [5, 6] has been defined as a process which consists of the conversion of digital images into high-dimensional data and the ensuing data mining to support clinical decision making. Its potential in providing accurate prognostic and predictive information has been highlighted by several studies published in the last five years across different cancer types [7, 8]. In HNSCC, radiomics and machine learning are still in its infancy, both in terms of predictive and prognostic value and treatment-related toxicity assessment.

In light of this rapidly evolving scenario, the present analysis sought to define the current state of the art on the prediction of radiation-induced side effects (RISEs) in HNSCC through radiomics and machine-learning applications. No previous systematic reviews have been published on this topic. The main purpose was to evaluate the potential of these modern approaches in the assessment of radiation-induced toxicities, which could support a tailored radiation treatment management.

## 2. Materials and Methods

### 2.1. Search Strategy and Study Selection

A systematic review according to the PRISMA methodology [9] was performed to answer to the following research question: “Is radiomics combined with machine-learning methods effective in predicting radiation-induced toxicity in head and neck cancer patients?”

A literature search via PICO (Population, Intervention, Comparison, and Outcome) to identify articles published in MEDLINE/PubMed and EMBASE was independently conducted by two authors until June 2019. Discrepancies in study selection were solved by consensus. The main inclusion criteria for study selection were the reporting of machine-learning models based on radiomic analyses in the considered clinical setting. All the specific inclusion and exclusion criteria for study selection and leading keywords which were used to identify studies in both databases are reported in Table 1. To identify more papers, the Boolean operator “OR” rather than “AND” was used to link the keywords “radiomics” and “machine learning.” No restrictions for publication years or type were applied for study identification. Furthermore, three separate searches were conducted on MEDLINE/Pubmed via PICO (https://pubmedhh.nlm.nih.gov/nlmd/pico/piconew.php) according to the “Outcome” keywords “radiation toxicity”, “radiation tolerance,” and “radiation injury,” respectively.

Only original articles edited in English were eligible for this analysis. Review articles, editorials, meeting abstracts, commentary, letters, or other forms of reports were excluded. In selected cases, the full-text of those retrieved papers has been also analyzed to identity additional references satisfying the inclusion criteria.

### 2.2. Data Extraction and Study Quality Assessment

General characteristics of the included studies (first author, year of publication, patients number, and organ at risk (OAR) considered, as well as image modality and radiomic features) along with articles main results and relevant statistical results (radiomic model performance) were extracted and tabulated. Four authors (two independently and two in concordance) assessed the methodological quality of the included studies using the Radiomic Quality Score (RQS) [10], a tool consisting of sixteen items which provide an indication of radiomics study quality.

### 2.3. Statistical Analysis

The overall score for each analyzed study was obtained by the algebraic sum of the single RQS items (see the supplementary materials for overall RQS scores assessed by each author). To take into account the interrater variability, the average and standard deviation values of the authors' RQS scores were calculated; finally, the mean RQS value of each study was reported as a percentage.

## 3. Results and Discussion

**Figure 1:**
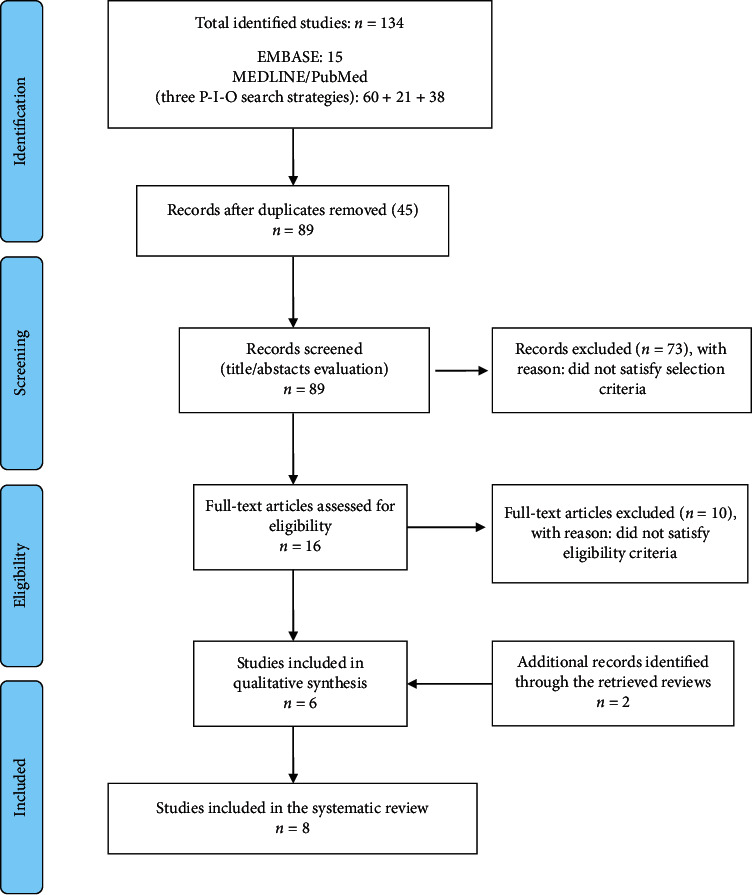
PRISMA flow diagram.

Among 134 identified studies, eight papers satisfying the inclusion criteria were specifically analyzed in this review. The PRISMA flow chart is shown in Figure 1. A summary of the main results of the included studies is reported in Table 2. Among the 8 included studies, two were identified as additional records through the retrieved reviews. Data on parotid glands, cochlea, masticatory muscles, and white brain matter were collected and analyzed.

### 3.1. Study Quality Assessment

Only the study by Van Dijrk [13] had an average RQS score ≥ 30% (50%), while 3 studies obtained a RQS score almost ≥ 25% (Abdollahi, Van Dijrk, and Gabrys) [14, 16, 18]. This finding suggests the lack of high-quality studies assessing the role of radiomics combined with machine learning in the prediction of radiation-induced toxicity in HNSCC cancer patients. We observed some interobserver discrepancies in the interpretation of specific RQS score items: the greatest were found for RQS score items “potential clinical applicability” and “open science and data,” even if other detected disagreements claim the need for more robust and easily interpretable methodological scoring systems for radiomic studies. Globally, higher standard deviations (4, 5) were observed in the studies by Leng, Abdollahi, and Pota [11, 14, 17].

### 3.2. Focus on Imaging Modality

In analogy with other findings reported for other cancer sites, computed tomography (CT) was the imaging modality analyzed in half of the selected articles [12, 14, 17, 18]. On the whole, the versatility, reproducibility, and integration into RT workflow make CT an ideal tool for multidimensional modelling. Through a longitudinal assessment of parotid glands, Scalco et al. [12] investigated the potential ability of quantitative imaging to predict parotid shrinkage, a well-known relevant issue in head and neck radiotherapy. With an interesting machine learning approach, Abdollahi et al. [14] explored the correlation between CT-related features of cochleas and the development of sensorineural hearing loss (SNHL) after treatment. When looking at very small ranges of interest (ROIs), the relatively low contrast resolution of CT is an intrinsic limitation and may lead to suboptimal segmentation, in particular if performed manually, as in this case. In addition, the significant association of the first- and second-order texture features with SNHL should be interpreted with caution, taking into account that cisplatin (a known ototoxic drug) was administered in less than two-thirds of cases and that pretreatment audiometry was not included in the proposed modelling. Pota et al. [17] further expanded Scalco's experience, by applying a novel artificial intelligence methodology (“likelihood-fuzzy analysis”). However, only 19 patients had complete information available for late xerostomia assessment, the primary endpoint in this work. In an innovative way, Gabrys et al. [18] were able to model the contribution of dose distribution to the contralateral parotid gland, parotid volume, and its asymmetry (or “eccentricity”). In particular, the authors were able to demonstrate that baseline small parotid glands (median volume of 9.5 mm^3^) and a steep right-left median gradient (1.7 Gy/mm) in contralateral parotid were significant risk factors for late xerostomia. Moreover, the study did not integrate a longitudinal (or “delta”) assessment; therefore, the impact of weight loss and parotid deformation throughout radiation could not be taken into account. Importantly, dose-gradient data may be more informative than mean dose itself when parotid glands are irradiated with a low range of dose, such as that commonly achieved with highly conformal intensity-modulated radiation therapy (IMRT) plans. Only one study in the present review is focused on the role of fluorodeoxyglucose positron emission tomography (FDG PET) [16]. In view of the possibility to capture information on tumor microenvironment, functional imaging may intrinsically have a greater predictive power than morphologic modalities. In this respect, cross comparing the work of Van Dijk et al. with a previous investigation from the same group [19], PET biomarkers were more informative than the CT ones. In particular, FDG PET hypermetabolism could better reflect the degree of activity within parotid glands and discriminate between fatty and nonfatty tissue in comparison with CT characteristics. High-intensity and texture features were associated with a lower risk of 12-month xerostomia; therefore, it could be hypothesized that hypercellularity within the gland may correlate with a lower radiosensitivity. On the other hand, outside of a prospective controlled trial, the dependence of semiquantitative PET features (such as SUV_max_) on scan acquisition parameters and anthropometric factors may be bias prone. In addition, the lack of follow-up scans to lend support to the authors' findings is a relevant limitation. The remaining 3 papers [11, 13, 15] were centered on magnetic resonance imaging (MRI) data. In a similar comparison with CT-based accuracy, the application of T1-weighted pretreatment MRI [13] was able to better detect the relationship between functional and nonfunctional parotid tissue and to improve the prediction ability of late xerostomia (AUC 0.83). The quantitative analysis of MRI intensity features is the most robust method to identify before treatment those patients at higher risk of toxicity. However, addressing MRI complex standardization is paramount. Taking all together, only one paper [13] reported on an external validation cohort and only 3 studies [11, 12, 17] can be considered in terms of “delta” radiomics. In this perspective, the analysis of intensity and texture features may contribute to the unresolved issue of replanning in HNSCC [20]. Overall, these imaging biomarkers may be the ideal candidate for parotid monitoring throughout treatment, since they mainly reflect variations in tissue organization. By scanning patients with nasopharyngeal cancer 3 times in planning position (first, second, and last weeks of RT course), Scalco et al. speculated that the early decrease into treatment of texture features (specifically of mean intensity and fractal dimension) may correlate with a rapid deterioration of glandular tissue, as known from pathologic data [21]. A similar approach was described by Pota et al., with CT acquisitions scheduled before RT, at the middle of treatment and after it. Taking into account that adaptive replanning is not supported by evidence for a routine use [22], a radiomics-based strategy may be highly advantageous also in terms of cost effectiveness. Parotid texture and volume features may represent a composite image biomarker with high sensitivity to assess tissue derangement throughout treatment. Finally, the work of Leng et al. [11] can be considered hypothesis-generating only, since the time-weighted monitoring of white matter injury with diffusion tensor magnetic resonance imaging (DT-MRI) cannot be considered predictive in the absence of a related clinical endpoint.

### 3.3. Xerostomia

Radiation-induced xerostomia is a major side effect for head and neck patients, and it has a considerable impact on quality of life [23]. Normal Tissue Complication Probability (NTCP) models that predict xerostomia are principally based on dose-volume parameters and baseline patient-rated xerostomia [24, 25]. However, there is a significant variance in predicting xerostomia with these models, so the improvement in the identification of patients at risk is crucial.

A better understanding of the mechanisms of radiation-induced xerostomia is necessary to advance towards more individualized treatments and improved sparing of normal tissues by dose optimization, with new radiation techniques such as proton therapy and MRI-guided radiation [13]. Radiomic features, such as shape, intensity, and texture characteristics extracted by images can contribute to the prediction of the disease response and survival [26]. However, the role of these image features to predict radiation-induced toxicities is not well explored.

Acute and late xerostomia symptoms are strongly associated with structural changes of parotid glands, which are in part related to parotid volume shrinkage during radiotherapy treatment [27]. It is known that when parotids shrink, they shift toward the midline, which is typically the high-dose region, thus a higher irradiation is received by the glands with respect to the planned dose [28]. Prediction of this volume shrinkage is, thus, relevant, since it allows personalized replanning strategies (adaptive radiotherapy) [29] which consider these anatomical variations, sparing the healthy parotid tissue from the highest dose regions [30].

In the study of Gabrys et al. [18], mainly based on machine-learning methods, the univariate analysis showed that parotid volume and dose shape features can be highly predictive of xerostomia. Patients with small parotid glands and steep dose gradients in the patient's right-left direction were significantly more likely to develop long-term xerostomia because of the shrinking of parotid glands during treatment toward the medial direction. The multivariate analysis highlighted the importance of other patient-specific (dose-independent) factors for the development of late xerostomia, such as parotid volume, parotid eccentricity, and the patient's sex. Females with small, elongated parotid glands were at higher risk of long-term xerostomia than males with large and round parotids. In many works, clinical and dosimetric parameters were considered possible predictors of the shrinkage process and xerostomia, such as age, body mass index, tumor location, planned dose to parotid glands, initial parotid glands volume, and overlap between parotid glands and lymph node metastases [31, 32], but the predicting power of models found by considering only these types of features can be improved using radiomic features extracted by imaging, suggesting that the presence of radiation-induced toxicity could also be explained by the structural properties of the glands. Recent investigations have suggested that parotid deformation may be related to complex structural and functional modifications [31]. Obviously, different types of imaging reveal different structural changes of parotids, due to the peculiarity of each imaging technique in distinguishing the various structural components of salivary glands. Scalco et al. [12] showed that there is a variation in the mean intensity of parotid glands on CT images during a RT course, suggesting a loss of acinar cells with a decrease of entropy due to an increase of adipose ratio in parotid during treatment. Another work compared parotids of normal subjects with parotids submitted to RT using ultrasound images [33]. The authors found an increase in tissue heterogeneity in post-RT subjects, with an increase in variance and entropy with respect to normal subjects. The latter two analyses were apparently in disagreement, but Yang's study based on ultrasound and not on CT images; in fact, the tissue of normal parotid glands, filled with serous acinar cells, provides uniform and highly reflective interfaces for the ultrasound beam. After RT, the loss of acinar cells in parotids leads to a more disorganized tissue organization, appearing in ultrasound images as a heterogeneous echographic pattern. Decrease in local entropy, seen with CT images, can be interpreted in the same way [12]. A study by Van Djik et al. [13] is based on MRI. MRI is superior in defining soft tissue contrast and, therefore, more accurate in differentiating fat from the parenchymal gland tissue [34]. RT can cause increased fat concentration in parotid gland during treatment (due to parenchymal changes determined by lipid infiltration), so radiomic features extracted by pretreatment MRI can increase the probability of predicting late xerostomia after radiotherapy [13].

The same group published another study [16], based on PET imaging, suggesting that patients with low metabolic parotid glands, quantified by features extracted by pretreatment FDG PET, were more likely to develop late xerostomia. This finding suggests that the nonfunctional (which can be fatty tissue) to functional tissue ratio is an important pretreatment characteristic to improve prediction of xerostomia. Moreover, high metabolic parotid glands could have more viable cells (parenchyma and/or stem cells) with more repair capability and/or could be less radiosensitive.

In the study of Pota et al. [17], the final parotid shrinkage rate was found to be correlated with 12-month xerostomia. Patients with low half thickness have lower probability of undergoing the problem of parotid shrinkage than patients with high half thickness. This means that patients of larger size are more at risk, and patients with low initial parotid volume have lower probability of parotid shrinkage than patients with high initial parotid volume. These results seem to be in conflict with other results [18], but this is not true if we consider (as showed previously) that xerostomia depends mainly on glandular structure and that radiomic features extracted by imaging sharply improve the predicting power of models based only on clinical and dosimetric parameters. For example, in 2015, Sanguineti et al. [35] showed that patients with rapidly shrinking parotids during the earlier part of treatment were those at higher risk of developing acute xerostomia; but the opposite is true: shrinkage during the first part of treatment predicts a higher rate of long-term recovery. In fact, acinar cell loss is the main cause of functional damage in human salivary glands after RT, and we previously found that parotid shrinkage during treatment is accompanied by a decrease in tissue density consistent with an increase in fat over glandular tissue. Even if a given dose of radiation would kill the same fraction of cells, the absolute damage would be higher for those glands with a lower baseline acinar component. On the other hand, there is a clear possibility that more sensitive patients (showing larger shrinkage) could experience a faster replacement of the acinar cells due to the activation of stem cells, efficiently recovering the gland functionality.

More recently, a confirmation of the potential improvement in xerostomia risk stratification by integrating baseline image features into predictive models, with the aim to ensure tailored HNSCC radiotherapy, has come from Sheikh's results [36], which suggested that baseline CT and MRI features may reflect baseline salivary gland function and potential risk for radiation injury. In 2020, Wilkie et al. [37] also showed that the addition of pretreatment parotid gland PET biomarkers improved a predictive model for late xerostomia over dose and pretreatment symptoms.

### 3.4. Other Toxicities

Chemoradiation can induce SNHL in HNSCC patients, and it could have a great impact on the patient's quality of life. Abdollahi et al. [14] integrated image features of the cochlea with clinical measures to improve the prediction of SNHL in HNSCC patients treated with chemoradiation: their results were obtained through a function of multiple CT imaging, dosimetric, clinical, and biological variables including radiomic features, age, sex, generalized equivalent uniform dose, radiation dose, and number of fractions, and chemotherapy. The results of this associations demonstrated that sex, generalized equivalent uniform dose, and concurrent chemoradiotherapy have a statistical impact on SNHL. Other variables are not associated with SNHL. Further analyses should be conducted using MRI radiomic features due to the major resolution for hearing structures obtained with MRI.

Trismus as RISE in HNSCC patients could be observed in up to 50% of all head and neck survivors. Mouth-opening limitation appears between three and twelve months after RT, and it produces eating and speech difficulties, with a considerable impact on patients' quality of life. Trismus after RT is a consequence of masticatory muscle contraction due to RT-induced fibrosis. Thor et al. [15] investigated an MRI approach to quantify radiation-induced masseter and medial and lateral pterygoids and temporalis muscle injuries applied to trismus. For these muscles, 24 textures from a T1-weighted MRI scan post-contrast were extracted with the aim to identify the related intramuscle intensity patterns muscles responsible for the radiation-induced trismus. The authors used univariate logistic regression to compare the muscle mean dose and textures between 10 cases and 10 control (ipsilateral muscles). The mean dose to the masseter and medial pterygoid related to the mean MRI intensity of these muscles could be a candidate predictor for trismus cases compared to controls.

The damage to the whole brain white matter (WM) in nasopharyngeal carcinoma patients after RT is due to blood vessel injuries that produce consequent ischemic necrosis. DT-MRI is the technology that can better evaluate the microstructural and morphological change of WM associated with RT. In the study by Leng et al. [11], DT-MRI, fibre bundle-/tract-based spatial statistics, and machine learning methods were used to study change in the whole brain white matter structure. After RT, patients were divided into three groups according to the stage of radiation brain injury: the acute reaction period, early delayed radiation period, and late delayed radiation group. The WM injury is a gradual and irreversible process located in the temporal lobe and bilateral cerebella, probably because these regions are near to the treated volumes. With the proposed machine-learning method, authors concluded that there was no observation of WM damage in the extensive brain region. After a period of progressive aggravation, the destruction of the whole brain can be gradually restored, due to the compensation and self-repair of the whole brain. These brain-discriminating WB regions could be used as biomarkers for clinical diagnosis of radiation brain injury.

### 3.5. Study Limitations and Future Perspectives

At the time of this review article, only few published reviews discussing the role of radiomics and machine-learning methods in HNSCC radiotherapy [38] were published; these previous analyses had a general focus on both adverse events and response/survival outcomes and reported the necessity of prospective, multicentric trials to prove the actual benefit of the use of these modern approaches in clinical practice.

A major limitation of the present analysis is the lack of large evidence from multiple high-quality radiomic studies assessing specific RISE. Indeed, only a limited number of published radiomic studies satisfied the inclusion criteria for our review, and heterogeneity in outcomes' assessment has been observed. Furthermore, only limited radiomic studies obtained high-quality RQS values due to observed limitations in internal consistency, reproducibility, clinical relevance, and applicability.

In addition, even if the RQS is a score system which supports the evaluation of quality level of radiomics studies, supplementary considerations are still required for both a comprehensive understanding of the radiomics process and a more accurate study quality assessment; these necessities are mostly due to potential variability in the interpretations of specific RQS items which could influence the interrater agreement in specific cases.

Because of all the aforementioned reasons, our findings should be carefully interpreted and several radiomic analyses from prospective clinical trials are encouraged for the validation of imaging biomarkers.

At the time of our search on the *clinicaltrials.gov* website, the NCT03294122 and NCT02489084 studies were assessing models, also based on image biomarkers analyses, to predict RISE in HNSCC, regardless of the primary tumor site. We specifically observed an emerging interest on the influence on RISE of the microenvironment (e.g., microbiota-host relationship and inflammatory markers), as well as on predictive models based on DNA profile assessment.

## 4. Conclusions

The radiomic analysis of images acquired during the diagnostic-therapeutic pathway of HNSCC patients may provide data relevant to improve predictive models for RISE. In selected cases of normal tissues exposed to radiations (e.g., parotid glands), the evaluation and integration into predictive models of baseline CT and MRI features and pretreatment PET biomarkers could be relevant for the evaluation and management of RISE; indeed, radiomics information should reflect baseline normal tissue's function and potential risks for late toxicity. Posttreatment images should support clinical findings and models' accuracy.

Nevertheless, at the time of this review, only limited studies seem to be useful for evaluating the potential of these modern approaches in the assessment of radiation-induced toxicities. Moreover, the radiomic studies which have been reviewed in this paper using a systematic approach provide preliminary data that require further validation to improve the decision-making processes. In this scenario, further studies using radiomics-based models and machine-learning applications with a large-scale validation system are encouraged.

## Figures and Tables

**Table 1:** Study selection criteria and research keywords according to the PICO model.

Selection criteria	Inclusion criteria	Exclusion criteria	EMBASE search via PICO	MEDLINE/PubMed search via PICO
P: population	Adults (age >18 years) affected by nonmetastatic HNSCC (nasopharynx; oral cavity; oropharynx; hypopharynx, larynx; nasal cavity; and paranasal sinus); salivary gland cancer	Pediatric patients (age < 18); non-HNSCC primary tumors; metastatic HNSCC cancer; and diagnosis of cutaneous squamous cell carcinoma or basal cell carcinoma of HNSCC	“Head and neck tumor”/exp OR “head and neck cancer”/exp	Head and neck tumor

I: intervention	Radiomics with artificial intelligence; radiomics-based machine-learning methods; and quantative radiographic phenotype analysis	Exclusion of radiomic analysis from the machine-learning method (exclusive analysis of biomarkers, genetic profiles, clinical data, etc.)	“Radiomics”/exp OR “machine learning”/exp	Radiomics OR machine learning

C: comparison	(Not explored)			

O: outcome	Radiation-induced toxicity; radiation-induced toxicity risk	Prediction of survival outcomes; local disease response; prediction of HPV-status or nodal status; and automatic contouring implementation	“Radiation toxicity”/exp OR “radiation tolerance”/exp OR'radiation injury'/exp	Radiation toxicity/radiation tolerance/radiation injury

**Table 2:** Summary of the main results of the selected studies. The table reports mean and standard deviation values of RQS which were attributed by authors to the included studies, along with a synthesis of the main results from each study (toxicity outcome's prediction according to the performed radiomics and machine-learning analyses).

Study	Outcome	Imaging modality	Radiomic features	OAR	Patients number	Results	RQS (mean ± standard deviation)	RQS (mean, percentage)
Leng et al. [11]	Radiation brain injury	Diffusion tensor imaging (DTI)-MR	Fractional anisotropy map (one of the most common DTI parameters)	Brain (white matter)	77	Machine learning in DTI-MR can aid the early recognition of white matter injury	8 ± 4	22.2

Scalco et al. [12]	Parotid shrinkage	CT	7 texture/fractal features (mean, variance, entropy, homogeneity, entropy S2, fractal dimension, and volume cc)	Parotid glands	21	A significant decrease in mean intensity (1.7 HU and 3.8 HU after the second and last weeks, respectively) and fractal dimension (0.016 and 0.021) was found. Discriminant analysis, based on volume and fractal dimension, predicted the final parotid shrinkage (accuracy of 71.4%)	-1 ± 2	2.8

van Dijrk et al. [13]	Late xerostomia (at 12 months after RT)	Pretreatment T1w-MR	21 intensity and 43 texture features	Parotid glands	Total 93 (68 + 25, from 2 centres)	90th intensity percentile values (that is, high fat concentrations) associated with higher risk of xerostomia	18 ± 2	50

Abdollahi et al. [14]	Sensorineural hearing loss (SNHL)	CT	490 extracted features	Cochlea	47	10 features are associated with SNHL (AUC 0.88)	10 ± 5	27.8

Thor et al. [15]	Trismus at 1 one-year post-RT	Posttreatment T1 *w* postcontrast MR	24 features	Masticatory muscles	20	Identification of mean dose/texture features candidate for trismus prediction	0 ± 1	0

van Dijrk et al. [16]	Late xerostomia (at 12 months after RT)	Pretreatment simulation FDG PET-CT	24 intensity and 66 texture features	Parotid glands	161	90th highest SUV values (high metabolic activity of the parotid gland) was associated with a lower risk of developing late xerostomia (xer12 m)	10 ± 1	27.8

Pota et al. [17]	Late xerostomia (at 12 months after RT)	CT	# Features	Parotid glands	37 (only 19 for xerostomia assessment)	Only preliminary data regarding the prediction of late toxicity, largely limited by the low sample size (*n* = 19)	4 ± 5	11.1

Gabrys et al. [18]	Late xerostomia (at 6–15 months and long-term toxicity outcome at 15–24 months after RT)	CT	# Radiomics and dosiomics features. Radiomic set: parotid shape (volume, sphericity, and eccentricity)	Parotid glands	153	Late xerostomia correlated with the contralateral dose gradient in the anterior-posterior (AUC = 0.72) and the right-left (AUC = 0.68) direction, whereas long-term xerostomia was associated with parotid volumes (AUCs >0.85), dose gradients in the right-left (AUCs >0.78), and the anterior-posterior (AUCs >0.72) direction	9 ± 1	25

## Data Availability

The data used in this study are given in the supplementary materials.

## Supplementary Materials

Supplementary Materials The data used in this study are given in the supplementary materials.
