# Supplementary material for: Investigation of Radiation-Induced Toxicity in Head and Neck Cancer Patients through Radiomics and Machine Learning: A Systematic Review
Source: J Oncol. 2021 Jun 9;2021:5566508. doi: 10.1155/2021/5566508 (PMC8211491; doi:10.1155/2021/5566508)
Supplement: Supplementary Materials — The data used in this study are given in the supplementary materials. [file 5566508.f1.pdf]

Study	1 Image Protocol Quality	2 Multiple segmentation	3 Phantom study	4 Tests-retests (imaging at multiple time-points)	5 Features reduction or adjustement multiple testing	6 Multivariate analyses with non radiomic features	7 Biological correlates	8 Cut-off analyses
Leng X (2019) [8]	1			1	3			1
Scalco E (2013) [9]	1			1	-3			1
Van Dijrk VL (2018) [10]	1				3	1		1
Abdollahi H (2018) [11]	1	1			3	1		1
Thor M (2017) [12]	1				-3	1		1
Van Dijrk VL (2018) [13]	2				3	1		1
Pota M (2017) [14]	1	1		1	-3	1		
Gabrys et Al. [15]					3	1		1

9 Discrimination statistics	10 Calibration statistics	11 Prospective study	12 Validation	13 Comparison to gold standard	14 Potential clinical utility	15 Cost-effectiveness analyses	16 Open science and data	Total score
2	2		-5					**5**
1			-5		2			**-2**
2	2		3	2	2			**17**
1	2	7	-5					**12**
2			-5		2		2	**1**
2	2		-5	2	2		1	**11**
	1		4		2		1	**9**
2	2		-5	2	2		1	**9**

Study	1 Image Protocol Quality	2 Multiple segmentation	3 Phantom study	4 Tests-retests (imaging at multiple time-points)	5 Features reduction or adjustement multiple testing	6 Multivariate analyses with non radiomic features	7 Biological correlates	8 Cut-off analyses	9 Discrimination statistics
Leng X (2019) [8]	1			1	3			1	2
Scalco E (2013) [9]	1			1	-3			1	1
Van Dijrk VL (2018) [10]	1		1		3	1		1	2
Abdollahi H (2018) [11]	1	1			3	1	1	1	1
Thor M (2017) [12]	1				-3	1		1	2
Van Dijrk VL (2018) [13]	1				3	1		1	2
Pota M (2017) [14]	1			1	-3	1			
Gabrys et Al. [15]					3	1		1	2

10 Calibration statistics	11 Prospective study	12 Validation	13 Comparison to gold standard	14 Potential clinical utility	15 Cost-effectiveness analyses	16 Open science and data	Total score
2	7	-5					**12**
		-5		2			**-2**
2		3	2	2			**18**
2		-5	2	2			**10**
		-5		2		2	**1**
2		-5	2	2		1	**10**
1		4		2			**7**
2		-5	2	2		1	**9**

Study	Image Protocol Quality	Multiple segmentation	Phantom study	Tests-retests (imaging at multiple time-points)	Features reduction or adjustement multiple testing	Multivariate analyses with non radiomic features	Biological correlates	Cut-off analyses	Discrimination statistics
Leng X (2019) [8]	1			1	3			1	2
Scalco E (2013) [9]	1	1		1	-3			1	1
Van Dijrk VL (2018) [10]	1		1		3	1		1	2
Abdollahi H (2018) [11]	1	1			3	1		1	2
Thor M (2017) [12]	1				-3			1	2
Van Dijrk VL (2018) [13]	1				3	1		1	2
Pota M (2017) [14]	1	1		1	-3	1			
Gabrys et Al. [15]					3	1		1	2

Calibration statistics	Prospective study	Validation	Comparison to gold standard	Potential clinical utility	Cost-effectiveness analyses	Open science and data	Total score
1		-5	2			2	**8**
1		-5	2			2	**2**
2		3	2			2	**18**
1		-5				2	**7**
		-5				2	**-2**
2		-5	2			2	**9**
1		-5					**-3**
2		-5	2			2	**8**

1 Image Protocol Quality

Voters for each subject 3
Total votes 24

nij=votes Allowable values

		0	1	2			
subjects	1	0	3	0		0	9
evaluated	2	0	3	0		0	9
8	3	0	3	0		0	9
	4	0	3	0		0	9
	5	0	3	0		0	9
	6	0	2	1		0	4
	7	0	3	0		0	9
	8	3	0	0		9	0
	Total	3	20	1			
	pj	0,125	0,833	0,042			
	pj squared	0,016	0,694	0,002			

Pe medium
0,7118056

Fleiss kappa per 1 Image Protocol Quality
k= 0,7108434

< 0	Poor agreement
0.01 – 0.20	Slight agreement
0.21 – 0.40	Fair agreement
0.41 – 0.60	Moderate agreement
0.61 – 0.80	Substantial agreement
0.81 – 1.00	Almost perfect agreement

	P medium
	0,916666667
	Pi
0	1
0	1
0	1
0	1
0	1
1	0,333333333
0	1
0	1

2 Multiple segmentation

Voters for each subject 3
Total votes 24

nij=votes — Allowable values

		0	1				
subjects	1	3	0			9	0
evaluated	2	2	1			4	1
8	3	3	0			9	0
	4	0	3			0	9
	5	3	0			9	0
	6	3	0			9	0
	7	1	2			1	4
	8	3	0			9	0
	Total	18	6	0			
	pj	0,75	0,25	0			
	pj squared	0,563	0,063	0			

Pe medium
0,625

Fleiss kappa per 2 Multiple segmentation
k= 0,5555556

< 0	Poor agreement
0.01 – 0.20	Slight agreement
0.21 – 0.40	Fair agreement
0.41 – 0.60	Moderate agreement
0.61 – 0.80	Substantial agreement
0.81 – 1.00	Almost perfect agreement

	P medium
	0,833333333
	Pi
0	1
0	0,333333333
0	1
0	1
0	1
0	1
0	0,333333333
0	1

3 Phantom study

Voters for each subject 3
Total votes 24

nij=voti — Allowable values

subjects evaluated 8

	0	1				
1	3	0			9	0
2	3	0			9	0
3	1	2			1	4
4	3	0			9	0
5	3	0			9	0
6	3	0			9	0
7	3	0			9	0
8	3	0			9	0
Total	22	2	0			
pj	0,917	0,083	0			
pj squared	0,84	0,007	0			

Pe medium
0,8472222

Fleiss kappa per 3 Phantom study
k= 0,4545455

< 0	Poor agreement
0.01 – 0.20	Slight agreement
0.21 – 0.40	Fair agreement
0.41 – 0.60	Moderate agreement
0.61 – 0.80	Substantial agreement
0.81 – 1.00	Almost perfect agreement

	P medium
	0,916666667
	Pi
0	1
0	1
0	0,333333333
0	1
0	1
0	1
0	1
0	1

Study	1 Image Protocol Quality	2 Multiple segmentation	3 Phantom study	4 Tests-retests (imaging at multiple time-points)	5 Features reduction or adjustement multiple testing	6 Multivariate analyses with non radiomic features	7 Biological correlates	8 Cut-off analyses	9 Discrimination statistics	10 Calibration statistics	11 Prospective study	12 Validation
Leng X (2019) [15]	1	0	0	1	3	0	0	1	2	2	0	-5
Scalco E (2013) [17]	1	0	0	1	-3	0	0	1	1	0	0	-5
Van Dijrk VL (2018) [11]	1	0	1	0	3	1	0	1	2	2	0	3
Abdollahi H (2018) [12]	1	1	0	0	3	1	0	1	1	2	0	-5
Thor M (2017) [19]	1	0	0	0	-3	1	0	1	2	0	0	-5
Van Dijrk VL (2018) [13]	1	0	0	0	3	1	0	1	2	2	0	-5
Pota M (2017) [16]	1	1	0	1	-3	1	0	0	0	1	0	4
Gabrys HS [14]	0	0	0	0	3	1	0	1	2	2	0	-5

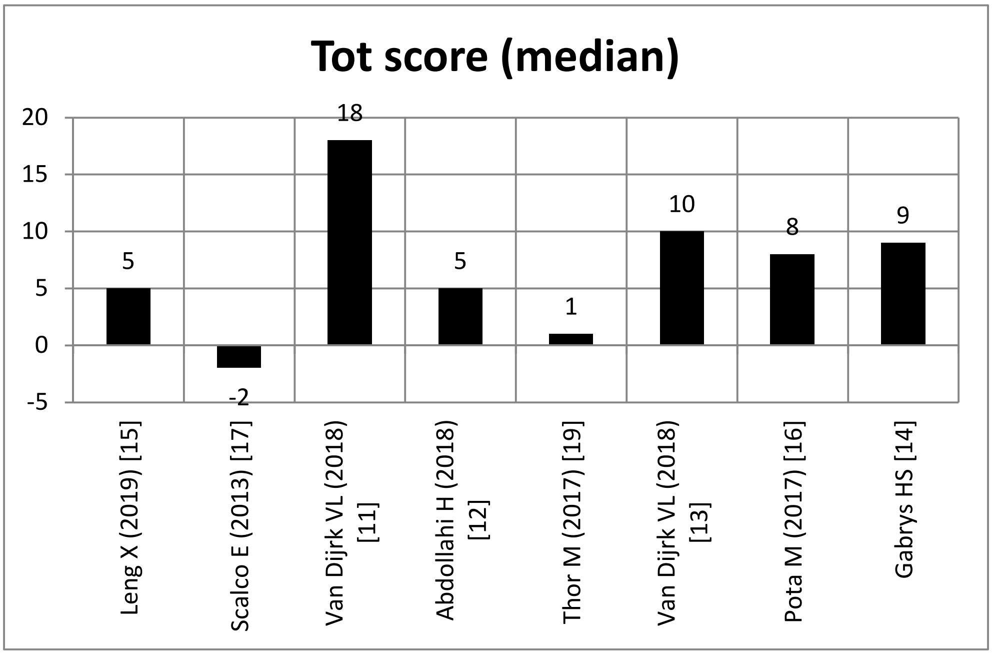

13 Comparison to gold standard	14 Potential clinical utility	15 Cost-effectiveness analyses	16 Open science and data	Tot score
0	0	0	0	5
0	2	0	0	-2
2	2	0	0	18
0	0	0	0	5
0	2	0	2	1
2	2	0	1	10
0	2	0	0	8
2	2	0	1	9

Study	1 Image Protocol Quality	2 Multiple segmentation	3 Phantom study	4 Tests-retests (imaging at multiple time-points)	5 Features reduction or adjustement multiple testing	6 Multivariate analyses with non radiomic features	7 Biological correlates	8 Cut-off analyses	9 Discrimination statistics	10 Calibration statistics	11 Prospective study	12 Validation
Leng X (2019) [15]	1	0	0	1	3	0	0	1	2	1,67	2,33	-5
Scalco E (2013) [17]	1	0,3333	0	1	-3	0	0	1	1	0,33	0	-5
Van Dijrk VL (2018) [11]	1	0	0,67	0	3	1	0	1	2	2	0	3
Abdollahi H (2018) [12]	1	1	0	0	3	1	0,33	1	1,33	1,67	2,33	-5
Thor M (2017) [19]	1	0	0	0	-3	0,67	0	1	2	0	0	-5
Van Dijrk VL (2018) [13]	1,33	0	0	0	3	1	0	1	2	2	0	-5
Pota M (2017) [16]	1	0,6667	0	1	-3	1	0	0	0	1	0	1
Gabrys HS [14]	0	0	0	0	3	1	0	1	2	2	0	-5

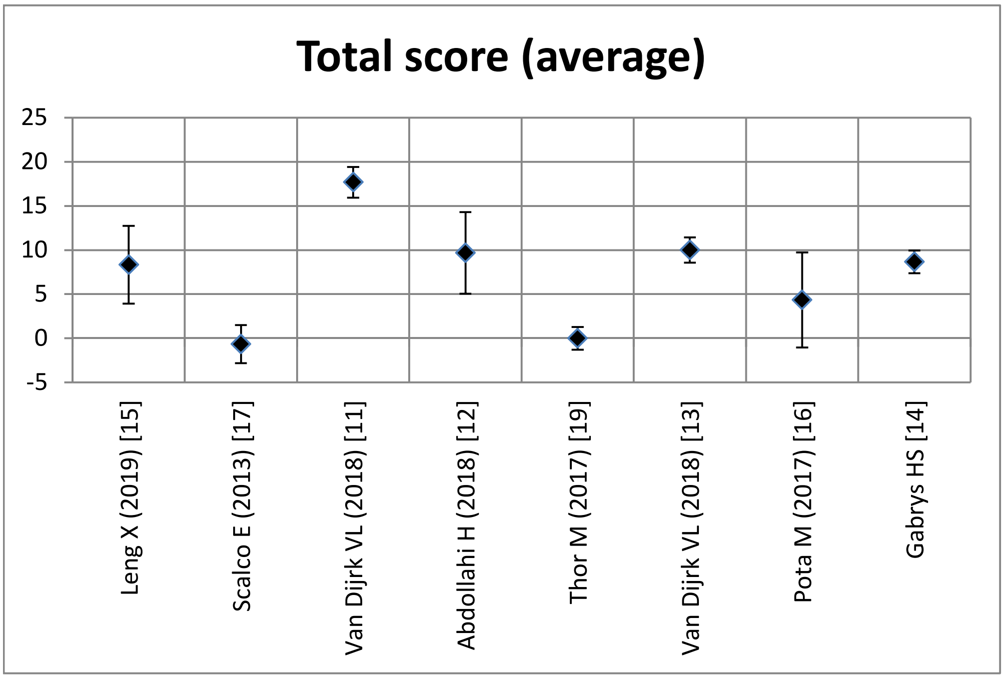

13 Comparison to gold standard	14 Potential clinical utility	15 Cost-effectiveness analyses	16 Open science and data	Tot score	sd on total
0,67	0	0	0,67	8	4
0,67	1,33	0	0,67	-1	2
2	1,33	0	0,67	18	2
0,67	0,67	0	0,67	10	5
0	1,33	0	2	0	1
2	1,33	0	1,33	10	1
0	1,33	0	0,33	4	5
2	1,33	0	1,33	9	1
